# Supplementary material for: Rapid Multiplex Real-Time PCR Method for the Detection and Quantification of Selected Cariogenic and Periodontal Bacteria
Source: Diagnostics (Basel). 2019 Dec 22;10(1):8. doi: 10.3390/diagnostics10010008 (PMC7168300; doi:10.3390/diagnostics10010008)
Supplement: Supplementary file 1 [file diagnostics-10-00008-s001.zip › Figure S1.pdf]

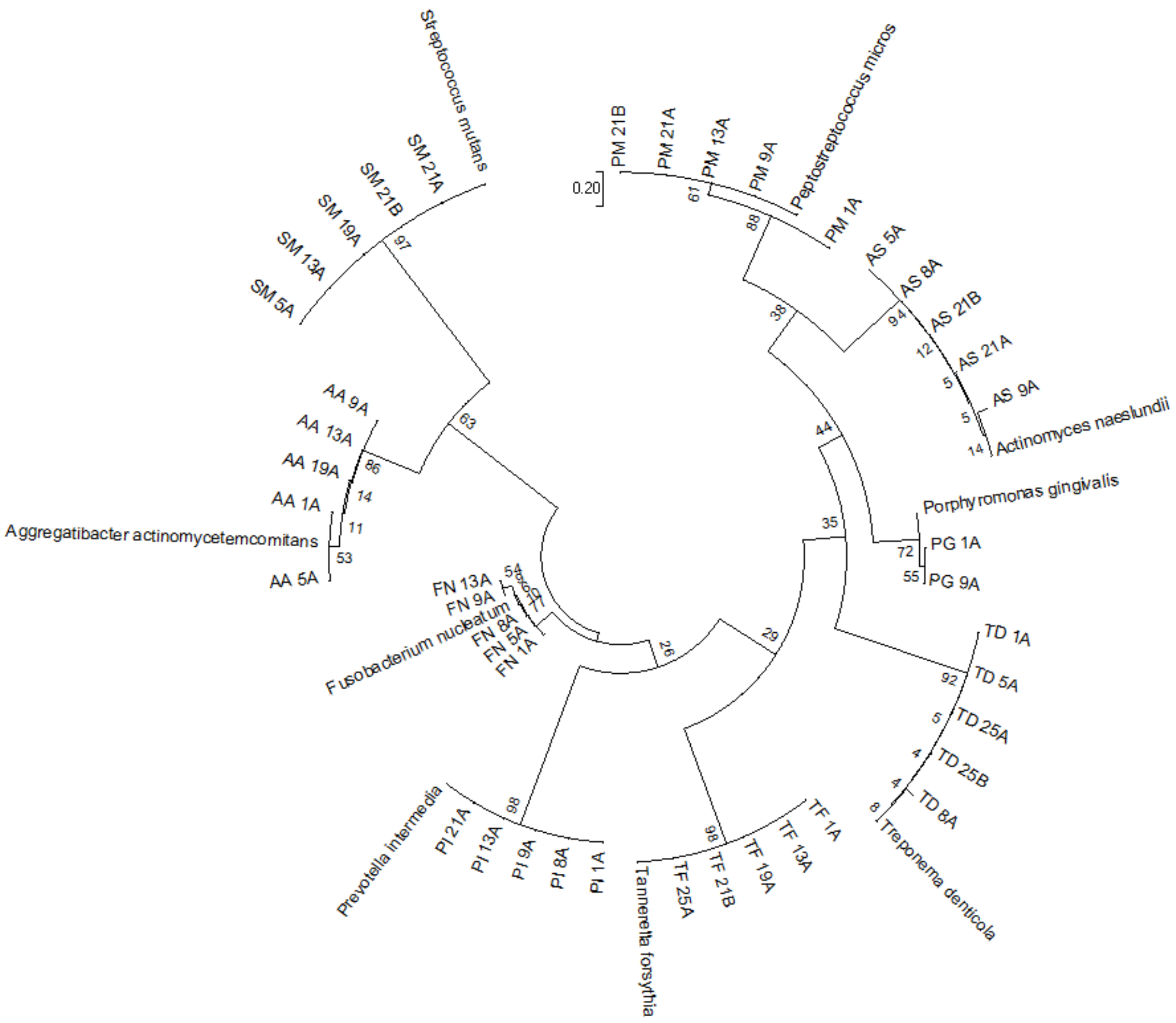

S1 Fig: Evolutionary relationships of taxa. The evolutionary history was inferred using the Neighbor-Joining method [1]. The evolutionary distances were computed using the Maximum Composite Likelihood method [2]. All positions containing gaps and missing data were eliminated. Evolutionary analyses were conducted in MEGA7 [3].

References

1. Saitou N, Nei M (1987) The neighbor-joining method: a new method for reconstructing phylogenetic trees. Mol Biol Evol 4:406–425. https://doi.org/10.1093/oxfordjournals.molbev.a040454

2. Tamura K, Nei M, Kumar S (2004) Prospects for inferring very large phylogenies by using the neighbor-joining method. Proc Natl Acad Sci USA 101:11030–11035. https://doi.org/10.1073/pnas.0404206101

3. Kumar S, Stecher G, Tamura K (2016) MEGA7: Molecular Evolutionary Genetics Analysis Version 7.0 for Bigger Datasets. Mol Biol Evol 33:1870–1874. https://doi.org/10.1093/molbev/msw054
